# Differential Digestive Stability of Food-Derived microRNAs: The Case of miR-30c-5p and miR-92a-3p in Polyfloral Honey

**DOI:** 10.3390/cimb46070443

**Published:** 2024-07-15

**Authors:** Diana Marisol Abrego-Guandique, Olubukunmi Amos Ilori, Maria Cristina Caroleo, Roberto Cannataro, Erika Cione, Paola Tucci

**Affiliations:** 1 Department of Health Sciences, University of Magna Graecia Catanzaro, 88100 Catanzaro, Italy; dianamarisol.abregoguandique@unicz.it (D.M.A.-G.); mariacristina.caroleo@unicz.it (M.C.C.); 2 Department of Pharmacy, Health and Nutritional Sciences, University of Calabria, 87036 Rende, Italy; lrilkn95a09z335g@studenti.unical.it (O.A.I.); paola.tucci@unical.it (P.T.); 3 Galascreen Laboratories, University of Calabria, 87036 Rende, Italy; rcannataro@nutrics.it; 4 Research Division, Dynamical Business & Science Society, DBSS International SAS, Bogota 110861, Colombia

**Keywords:** functional food, dietary microRNA, INFOGEST, *in vitro* digestion

## Abstract

Dietary microRNAs (miRs) represent a new area in food science. Although they have been found in many foods, including honey, more research is needed about their stability and fate during digestion. Hence, this study aimed to analyze the digestive stability of two selected miRs in honey. We extracted miR-92a-3p and miR-30c-5p from pasteurized and unpasteurized forms of polyfloral honey using two different methods and kits: a column-based manual method and a phenol-free semi-automated magnetic-bead-based method. The latter option was used for the subsequent analysis of samples according to the INFOGEST static *in vitro* digestion protocol. Also, the honey samples were examined for exosome-like particles using dynamic light scattering. Although the expression levels of both miRs were significantly lower following intestinal digestion, we found a difference in the resilience of the miRs to gastrointestinal conditions, with miR-30c-5p being relatively stable compared to miR-92a-3p following digestion, regardless of the honey’s pasteurization treatment. Moreover, there was marked heterogeneity in the extracellular vesicle profile of the pasteurized sample. We identified the presence of two broadly conserved miRs in honey: miR-92a-3p and miR-30c-5p. Despite honey exhibiting high digestibility, miR-92a-3p was less resilient than miR-30c-5p, demonstrating considerable resistance under gastrointestinal conditions. Although further research is needed, the results obtained from this study may represent a starting point for utilizing honey as a source of exogenous miRNAs for preventive strategies and more “natural” treatments.

## 1. Introduction

MicroRNAs (miRs) are short, non-coding RNA molecules that play critical roles in regulating gene expression at the post-transcriptional level [1]. Indeed, by binding to messenger RNA (mRNA) molecules and either degrading them or inhibiting their translation, they effectively control the levels of proteins produced within cells [2,3,4,5]. Moreover, miRs have gained attention for their importance in food science and nutrition, as they have been detected extensively in various dietary sources, including fruits and vegetables, meat and its products, milk (including human breast milk) and other dairy products, and honey [6,7,8]. Although miRs have been long considered to be synthesized endogenously and there has been little study of miR cross-species transportation, recent studies have revealed that miRs have exogenous roles as well [9]. Studies have demonstrated that plant miRs can survive exogenously in a mature form in animals [10]. The most important evidence of these dietary miRs was the detection of endogenous mammalian miRs in breast milk, with different contents from mother to mother [6,7,8]. These miRs might function as a nutritional component, being transferred from the mother to the infant through breastfeeding, thus providing benefits to the infant by helping shape their immune system and gut microbiome [11]. They can be players in the development of oral tolerance [12]. Certain dietary miRs have been shown to play a role in preventing diseases [13]. For instance, plant-based miRs can modulate inflammation and immune responses [14], potentially reducing the risk of chronic diseases like cancer and cardiovascular conditions.

Moreover, miRs in food can affect the composition and function of the gut microbiota, which, in turn, influences overall health [15]. They help maintain a balanced gut flora, which is crucial for digestion, nutrient absorption, and immune function. As a result, several investigations have been conducted, which have identified myriad miRs in many animal and plant food products. In 2017, Gismondi et al. described for the first time the presence of miRs in honey [16]. Honey is not just a sweet treat but also offers several nutritional benefits that make it a valuable addition to the everyday diet, as demonstrated by its global market share, amassing USD 8.66 billion in 2023 [17]. Honey and its products significantly impact alternative medicine through their immunomodulatory properties [18,19,20]. Other studies identified the presence of two broadly conserved miRs in honeybees: miR-92a-3p and miR-30-5p [21,22]. These miRs have been identified as being differentially expressed in different disease states [23,24,25]. Meanwhile, hsa-miR-30c-5p was negatively correlated with the influenza A virus [26] and showed tumor-suppressive properties [27], and members of its family are abundant in breast milk [28].

While miRs have garnered attention for their stability under different conditions and their role in biological processes, their survival and persistence in animal gastrointestinal tracts remain unexplored [29]. In principle, dietary miRs undergo processing, absorption by the gastrointestinal tract, delivery by the circulatory system, and transport into multiple tissues before exerting their functions. Although studies have demonstrated selective resistance to gastrointestinal digestion among dietary miRs, with some exhibiting higher stability than others, there is little information relating to the fate or survival of the dietary miRs under different conditions, especially simulated gastrointestinal conditions, which are crucial for understanding their potential as exogenous regulators of biological processes. The miR-92a family is broadly conserved in many species, with a highly significant E-value and perfect percent identity between the sequence of humans and honeybees for this miR [30]. Additionally, miR-30-5p is broadly conserved, and honey could contain miRs from itself as sources beyond the plant kingdom and its producing species [31], which is a familiar venture. Despite honey being a subject of intense research, more is needed regarding its contribution to diet-related miR transfer from A. mellifera to humans. In the same vein, inadequate attention has been given to the stability of miR-30c-5p and miR-92 in foods, especially in honey, following digestion. Thus, using *in vitro* digestion models, this work aimed to examine whether miR-30c-5p and miR-92a-3p were stable after simulated digestion. Notably, these two miRs have immunomodulatory properties [23,24,25,32,33]. Although these models were designed to study the digestibility, structural alterations, and release of food and drug constituents under simulated gastrointestinal conditions, they can also be used to evaluate the stability of miRs [34,35,36].

## 2. Materials and Methods

### 2.1. Sample Collection

Pasteurized and unpasteurized honey samples were collected and obtained directly via apiculture in the central and northwest zone of Calabria, in Southern Italy. Four honey samples were collected for each zone at the end of October 2023 (Supplementary material Figure S1). Unlike milk, honey is not pasteurized for food safety reasons but for quality purposes since the process delays granulation. The samples were held at room temperature and analyzed as described below. In this study, we evaluated only the polyfloral variety, also known as wildflower honey, derived from various plant species’ nectar. It reflects the health of the surrounding ecosystem, indicating a rich biodiversity of flowering plants. Moreover, supporting polyfloral honey production encourages sustainable agricultural practices and the conservation of natural habitats.

### 2.2. In Vitro Digestion of Honey Samples

The samples were subjected to simulated gastrointestinal digestion according to the INFOGEST protocol and as described by Minekus et al. [37] and Brodkorb et al. [38]. Unless otherwise stated, all enzymes and reagents used in the digestion were purchased from Merck (Darmstadt, Germany) and Lipolytech (Marseille, France).

#### 2.2.1. Pre-Digestion Procedures

The pepsin activity of porcine pepsin (Sigma-Aldrich, St. Louis, MO, USA) or Rabbit Gastric Extract (RGE) (Lipolytech, Marseille, France) was determined based on the formation of TCA-soluble tyrosine peptides when pepsin acts on hemoglobin as described by Brodkorb et al. [38]. The activity of trypsin in porcine pancreatin (Sigma-Aldrich, St. Louis, MO, USA) was similarly assayed as portrayed by Brodkorb et al. [38], preceded by a solubilization procedure detailed in a previous study [39]. The bile acid concentration in bovine bile (Sigma-Aldrich) was determined using the Bile Acid Assay Kit (Sigma-Aldrich, St. Louis, MO, USA) and according to the manufacturer’s specifications. Gastric lipase activity in RGE was measured following the method detailed in Grundy et al. [40] with modifications. Briefly, 0.5 mL of Glyceryl tributyrate (Sigma-Aldrich, St. Louis, MO, USA) was mixed with 14.5 mL of assay solution composed of 150 mM NaCl, 2 mM CaCl_2_, and 1 mM Bovine Serum Albumin (BSA) at pH 5.5 to form an emulsion. The mixture was stirred (Velp Scientifica ARE, Usmate Velate, MB, Italy) until the pH and temperature were stable at 5.5 and 37 °C, respectively. Then, 50, 100, or 200 µL of 1 mg/mL enzyme solution was introduced into the mixture and the pH was maintained at 5.5 during the subsequent 10 min by pipetting 0.1 N NaOH, using low retention tips, in volumes of 2–4 µL. The specific activity of gastric lipase was computed using the total volume of 0.1 N NaOH according to Grundy et al. [40].

#### 2.2.2. INFOGEST Static *In Vitro* Gastrointestinal Digestion

**Scheme 1:**
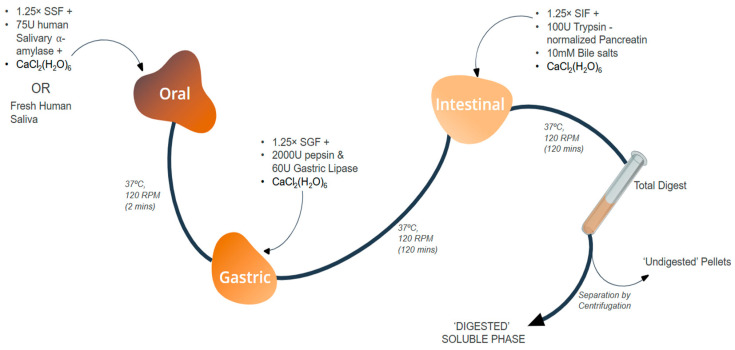
INFOGEST protocol procedure.

The *in vitro* digestion of the samples was performed according to the harmonized INFOGEST protocol described by Minekus et al. [37] and Brodkorb et al. [38] with some modifications. The 1.25X simulated salivary fluid (SSF), gastric fluid (SGF), and intestinal fluid (SIF) were prepared. No assay was performed to determine the activity of salivary amylase; hence, the activity recommended by the manufacturer was used during the digestion. The quantity of enzymes and bile used were computed using the designated tool on www.proteomics.ch/IVD (accessed on 15 January 2024), depending on the number of samples. The 3 phases that constituted the digestion are summarized in Scheme 1: in the oral phase, 1 g of the sample (honey) was mixed with 1 mL of pure human saliva [41] in a pre-weighed 50 mL Falcon tube, vortexed, and incubated at 37 °C for 2 min. Alternatively, the sample was mixed with 0.8 mL of 1.25X SSF, 0.1 mL of human salivary α-amylase (Sigma-Aldrich, 75 U), and 1.5 mM CaCl_2_(H_2_O)_6_ and made up to 2 mL with Milli-Q water before incubation. Subsequently, in the gastric phase, 1.6 mL of 1.25X SGF (pH 3), 0.15 mM CaCl_2_(H_2_O)_6_, 0.1 mL of porcine pepsin solution (2000 U), and 0.1 mL of RGE solution (60 U) were added, and the mixture made up to 4 mL with water. This was incubated at 37 °C for 120 min while being agitated. Thirty minutes before the end of gastric digestion, the pancreatin suspension was prepared as described previously [38] by dispersing pancreatin in SIF, vortexing, and sonicating at 130 W for 5 min before centrifuging at 2000× *g* for 5 min after which the supernatant was collected. Following gastric digestion, 1.7 mL of 1.25X SIF (pH 7), 0.5 mL of bile (10 mM), 0.6 mM CaCl_2_(H_2_O)_6_, and 1 mL of pancreatin (100 U Trypsin) suspension were added and the volume made up to 8 mL with water. This mixture was incubated at 37 °C for 120 min while being agitated. Digestion was halted by heating at 85 °C for 15 min. Each digestion was carried out in parallel with blank digestion, i.e., 1 mL of water in place of honey. Also, digests were obtained at the end of each of the three phases (oral, gastric, and intestinal) for each sample. To separate the soluble phase, 2 mL of the digest was centrifuged at 5000× *g* for 15 min at 4 °C, and the supernatant was transferred into new tubes. This was stored at −20 °C until further analysis. From the weight of the total digest and soluble fraction, the percentage of the soluble/digestible fraction was determined.

### 2.3. Characterization of Exosome-like Nanoparticles in Honey

Honey was examined for extracellular vesicles (EVs) based on Dynamic Light Scattering (DLS). DLS was performed using a Malvern Zetasizer Pro (Malvern Instruments, Malvern, UK) with ZS XPLORER software (version 3.0). The honey sample was prepared by mixing with water in a ratio of 1:1 m/v, filtered (1 mL of HSW 2-part dispenser with 0.45 µm 13 mm PVDF filter), and diluted with water at a ratio of 1:10. The diluted sample was pipetted into a disposable folded capillary cell (DTS1070 cells) and the measurement was taken with the equipment at 25 °C and an equilibration time of 60 s. From the measurement, the Z-average diameter and polydispersity index were obtained.

### 2.4. Total RNA Extraction from Honey and Its Digests

Unless otherwise indicated, all reagents used in miRNA isolation and amplification and the instrumentation were obtained from Thermo Fisher Scientific. Two methods of extraction, manual and semi-automated, were employed. RNA from digests and undigested samples were extracted in triplicates and duplicates, respectively.

#### 2.4.1. Column-Based Extraction (Manual)

A column-based kit, which contained phenol, was used in the initial extraction of total RNA (including small RNAs) from the undigested polyfloral honey. The reagents used were supplied along with the miRNeasy serum/plasma kit obtained from Qiagen (Hilden, Germany). A 280 mg honey sample was dissolved in 200 µL of RNase-free water and was incubated at room temperature for 5 min with 1 mL of Lysis reagent. The subsequent steps of the extraction procedure followed the miRNeasy serum/plasma kit quick-start protocol.

#### 2.4.2. Semi-Automated Extraction

Total RNA, including small RNAs, was extracted using MagMAX mirVana Total RNA Isolation Kit (Thermo Fisher Scientific, Waltham, MA, USA) on a KingFisher Duo Prime Magnetic Particle Processor (Thermo Fisher Scientific, Waltham, MA, USA). Both the pasteurized and unpasteurized honey samples of the polyfloral honey were extracted in parallel with their digests. Undigested honey samples were prepared by mixing 280 mg with 200 µL of RNase-free water. Then, 100 µL of this mixture or the soluble phase of the digests was added to the wells in the second row of a 96-Deep-Well Plate alongside 45 µL of Digestion Buffer and 5 µL of Proteinase K. Subsequent steps of the extraction procedure followed the instructions from the Applied Biosystems MagMAX mirVana Total RNA Isolation Kit user guide (Thermo Fisher Scientific, Waltham, MA, USA). RNA from digests and undigested samples was extracted in triplicates and duplicates, respectively.

### 2.5. Total RNA Quantification

The quantity, as well as the purity, of the extracted RNA (including miRNAs), was measured using a Thermo Scientific NanoDrop One instrument (Thermo Fisher Scientific, Waltham, MA, USA). The equipment was blanked with RNase-free water and 2 µL of the extracted RNA was loaded onto the pedestal. Measurements were taken in duplicates, and the quantity of the total RNA in ng/µL was recorded with the quality parameters (A260/A280 and A260/A230).

### 2.6. qRT-PCR of Honey and Its Digests

The extracted miRs were amplified and quantified using the TaqMan Advanced miRNA cDNA Kit (Thermo Fisher Scientific, Waltham, MA, USA), with the Primers used equally purchased from Applied Biosystems. The isolated RNAs were normalized to 5 ng/µL, 2 µL of which was used to start a Poly(A) reaction as per the manufacturer’s instruction (Applied Biosystems™ 2720 Thermal Cycler). Following the TaqMan Advanced miRNA user guide, the reaction product obtained was used in the subsequent adaptor ligation, reverse transcription, and complementary DNA (cDNA) synthesis reactions. The PCR reactions were set up using 5 µL of the diluted cDNA (1:10 with RNAse-free water) with 10 µL of 2X TaqMan Advanced Master Mix, 4 µL of RNase-free water, and 1 µL of 20X TaqMan Advanced miRNA Assay containing the primers in each well of a 96-well reaction plate. The PCR analyses were carried out using the miR-30c-5p 478008_mir (5′–UGUAAACAUCCUACACUCUCAGC–3′) and miR-92a-3p 47827_mir (5′–UAUUGCACUUGUCCCGGCCUGU–3′) primers. Compliance with the Minimum Information for the Publication of Real-Time Quantitative PCR Experiments (MIQE) guidelines [42] was provided, and all experiments were performed in triplicate.

The plate was centrifuged at 4000 rpm for 5 min to collect the contents at the bottom of the well. The real-time (RT) PCR was set up and run on a QuantStudio™ 5 system (PCR System, Applied Biosystems™, Madrid, Spain) using the following cycling conditions on a fast-cycling mode with a comparative Ct type: one cycle of 95 °C for 20 s (enzyme activation) and 40 cycles each of 95 °C for 1 s (denaturation) and 60 °C for 20 s (annealing and extension). The qRT-PCR was repeated for another independent semi-automated extraction, resulting in duplicates. The Ct values obtained were inverted (ICt) by subtracting from 40 and then averaged as described in clinical samples [43].

### 2.7. Analysis and Visualization

The results were analyzed using IBM SPSS Statistics (version 27.0). Descriptive statistics of means and/or standard deviations (SD) were used to present the results of RNA concentrations and miRNA expression. The Shapiro–Wilk Normality test was used. If the data were normally distributed, an independent *t*-test was, therefore, used to compare the means between two groups (where necessary) at a 5% level of significance. Results were visualized using Graph Pad Prism 10.2.3 for Windows (GraphPad Software).

## 3. Results

### 3.1. Honey Digestibility

The digestibility of honey, estimated from the mass balance of the soluble phase of the honey digests across the three phases of digestion, was reasonably high (being comparable to blank digestion), ranging from 92 to 99% (Table 1).

### 3.2. Variation in Total RNA Yield According to Methods of Extraction

The use of a solid-liquid phase in-column RNA purification method yielded total RNA concentrations between 0.64 and 0.51 µg/g (Table 2). The RNA quality obtained here is relatively low with the manual method. This is expected, as the quality parameters A260/A280 and A260/A230 may be unusable when the concentration per µL of the extract is less than 10 ng/µL.

We considered a phenol-free extraction approach to improve purity. As a result, the total RNA yield per gram sample (Table 2) increased greatly. The improved isolation yield in the present study is mostly related to the isolation technology (binding beads) and the instrumentation, which limits human error during extraction and increases elution volume. Hence, extracts from the intestinal digests of honey exhibited optimal purity since they have higher RNA concentrations and a higher dilution ratio to honey, as shown in Table 3.

### 3.3. Vesicles Characterization of the Polyfloral Honey

**Figure 1:**
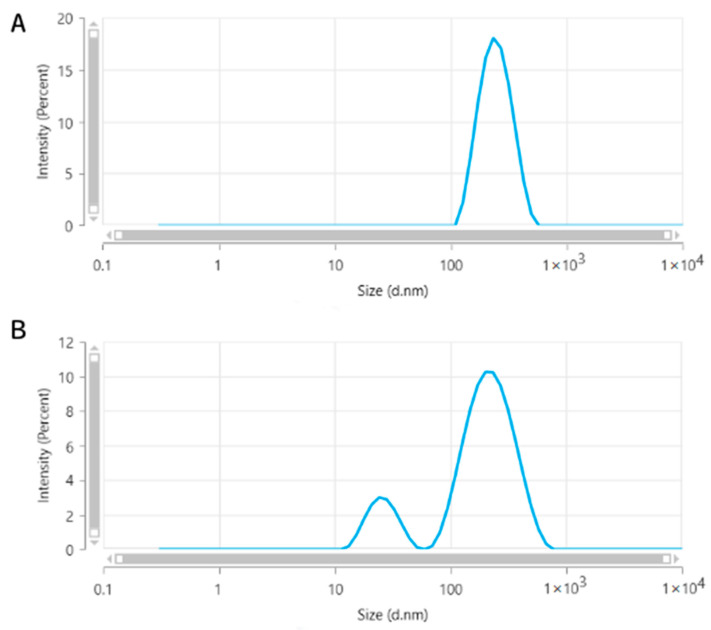
Dynamic Light Scattering Characterization of Exosome-Like Nanoparticles (ELNs) in Polyfloral Honey. (**A**) Unpasteurized honey, Z-Average—195.9, mean peak—249.7; (**B**) Pasteurized honey, Z-Average—113.3, 1st mean peak—26.28, 2nd mean peak—236.9.

The possibility of differences in miR packaging prompted the examination of honey for potential carriers (as vesicles) using a very simple method of particle size measurement (Figure 1A).

The unpasteurized honey sample exhibited relative uniformity (polydispersity index = 0.302) in the size of its particle distribution, averaging 196 nm in diameter. In Figure 1, after the thermic treatment is present, there is a first mean peak at 26.28 nm and a second mean peak at 236.9 nm (Figure 1B).

### 3.4. Selective Digestive Stability of miR-30c-5p and miR-92a-3p

Both miR-30c-5p and miR-92a-3p are highly conserved miRs, we first confirmed the presence of both miRs in honey using isolates of the column-based extraction, and then, the Ct values obtained were compared with those of the semi-automated phenol-free extraction method (Table 4).

**Figure 2:**
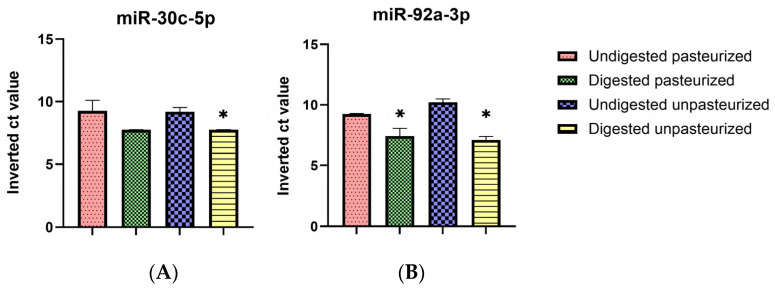
Inverted Ct Values of miR-30c-5p (**A**) and miR-92a-3p (**B**) in polyfloral honey and its digest. Bars are the means of 3 independent extractions. * Significantly lower at *p* < 0.05 (independent *t*-test). miR-30c-5p—unpasteurized, *p* = 0.011 (combined, *p* = 0.008). miR-92a-3p—pasteurized, *p* = 0.038; unpasteurized, *p* = 0.005 (combined, *p* < 0.001).

Then, to better visualize the stability after digestion, the ICt value was used. The ICt of the honey digests must exceed the corresponding blank digest to be considered “detectable” post-intestinal digestion; the average Ct for miR-30c-5p and miR-92a-3p in the blank digests were 32.34 and 31.08, respectively, translating into inverted Ct values of 7.66 and 8.92. The ICts of miR-30c-5p in the honey digests exceeded this, hence deeming miR-30c-5p “detectable” after intestinal digestion. On the other hand, ICts of miR-92a-3p in the digests were lower than the 8.92 in the blanks, rendering it “undetectable”. The results obtained indicated differing stability of the miRs under gastrointestinal conditions. Even though the expression levels of the miRs significantly decreased following gastrointestinal digestion, the current study demonstrates that miR-30c-5p is relatively stable (Figure 2A), compared to miR-92a-3p (Figure 2B).

## 4. Discussion

Honey is a supersaturated solution of sugars, containing about 69% monosaccharides, 10% disaccharides, and oligosaccharides [44], putting total sugar composition at approximately 80% irrespective of the variety [45,46,47]. The nature of the sugar present in honey makes it a readily available energy source with no need for extensive digestive enzyme activity.

The percentage of soluble fraction increased, although slightly, from one phase of digestion to the other, and this coincided with increased dilution afforded by the simulated digestive fluids. Hence, the increased digestibility in this case may be as related to the dilution of the saturated sugar solution as it is to the enzymatic breakdown of the disaccharides contained. This is quite prominent when samples digested with human saliva are compared against those in which simulated salivary fluid (SSF) was used (Table 1). Although both showed high honey solubility or digestibility, digests with saliva have a marginally lower soluble fraction compared to those with SSF. Human saliva offers a more viscous consistency owing to the presence of mucosal glycoproteins, and despite having about 30 proteins with catalytic properties [47], the soluble/digestible fractions of digests in which human saliva was used were still lower when compared to those involving SSF. This may be due to the necessity of the solvent capacity of water, which is expected to be higher in SSF, being less viscous [48,49]. Digestively, proteins are generally less than 1% in honey. Hence, the enzyme-to-substrate ratio during digestion will allow the nearly total breakdown of the little protein present. There was no notable trend in the digestibility of honey according to pasteurization in this study. The use of the mass balance of soluble phases of digests, coupled with the method of the inhibition of enzymatic reactions (usually heat-shock treatment), made identifying differences in the digestibility of pasteurized and unpasteurized honey unfeasible. The use of a solid-liquid phase in-column RNA purification method yielded total RNA concentrations between 0.64 and 0.51 µg/g, which is low when compared to those obtained by Gismondi et al. [16], who also used a total RNA extraction kit manually and reported 1.42 ng of RNA per mg honey sample. However, the same study yielded a value within the range of the current study (0.68 ng/mg) when another extraction method was used. Since the extraction method employed may impact the yield and purity of extracted RNA [50], we considered a phenol-free extraction approach to improve purity. As a result, the total RNA sample yield per gram increased greatly beyond those reported by Gismondi et al. [16] when they used a total RNA kit for apple honey. The improved isolation yield in the present study is mostly related to the isolation technology (binding beads) and the instrumentation, which limits human error during extraction and increases elution volume. Hence, extracts from the intestinal digests of honey exhibited optimal purity since they have higher RNA concentrations and a higher dilution ratio to honey.

The particle size measurements obtained here are slightly higher than those reported by Schuh et al. [51], who found a mean particle size of 186 nm, although the most occurring nanoparticle size was much lower in diameter (172 nm compared to 250 nm in this study). Differences in instrumentation employed and the geographical origin of the sample may be responsible for this disparity. Chen et al. [52] observed nanoparticle-like vesicles with an even lower diameter range (120–180 nm). Since they used a monofloral honey variety, the botanical origin (floral source) may be the distinct factor making these studies less comparable. All these studies, together with the current one, found particles with a range of diameters suitable for consideration as small to intermediate shed microvesicles (sMVs) [53,54]. The sMVs are similar to exosomes; however, they are larger in diameter than the latter, although there are proposals relating to differences in their origin [54]. Although the Zetasizer software (version 3.0) we used provided insight into the size and number of different EV populations in the samples, further analyses could be followed up in subsequent studies to determine the specific types of EVs present.

Honey can be consumed pasteurized or not. Usually, the pasteurization of honey is made to delay granulation. Both miR-30c-5p and miR-92a-3p are highly conserved miRs detected in many animal species and food products such as fish, chicken, and eggs [55,56]. Although the presence of miR-92a-3p was previously identified in honeybees’ brains [22], we first confirmed the presence of both miRs in honey using isolates of the column-based extraction, and then, the Ct values obtained were compared with those of the semi-automated phenol-free extraction method. The Ct values of the miRs obtained from both cases were comparable with those of some broadly conserved miRs found in polyfloral honey observed by Smith et al. [31], who reported Ct values ranging from approximately 29 to 35. Also, subjecting honey to thermal processing, such as pasteurization, did not exert any significant depreciation of the miRs, thereby confirming the significant stability of miRs under such conditions [57]. Since the expression level of miR-92a-3p in the column-based extracts was lower, the semi-automated RNA isolation method was considered for the qRT-PCR of the honey alongside its digests. The Ct values of the two miRs in the undigested honey here were similar; hence, it would not be astute to consider differences in the expression levels of the nucleic acids in the honey before digestion as a plausible reason for this selective stability. However, Gareev et al. [58] suggested that the differences in the packaging and the homogeneity of the forms of extracellular-existing miRs, lncRNAs, and circRNAs may influence their properties, which include structural integrity and biological functions. On the other hand, the pasteurized form exhibited more heterogeneity (polydispersity index = 0.576). In addition to the range of particles detected in the unpasteurized form, there was a range of particles with a smaller diameter, the average of which (26 nm) may be considered a little less to fit into exosomal size properties [34,35,36]. Hence, it is possible to suppose that the pasteurization process has destabilized the larger nanoparticles in the honey sample. It is not certain whether this phenomenon has been pointed out in a previous study, although Mu et al. [59] observed similar behavior under simulated gastrointestinal conditions. However, these results contrasted with the ones reported by Schulz et al. [60] and Cheng et al. [61], both of whom demonstrated the stability of isolated exosomes to heat over long periods. The relevance of examining the stability of EVs in situ must be considered as the isolation process might be too selective to include the degraded vesicles as part of the extracellular vesicle profile [53,62]. However, this did not exert any apparent difference in the stability of the examined miRs, since a relatively small percentage (17%) of the larger nanoparticles appeared to be affected.

The average Ct for miR-30c-5p and miR-92a-3p in the blank digests were 32.34 and 31.08, respectively, translating into inverted Ct values of 7.66 and 8.92. The ICts of miR-30c-5p in the honey digests exceeded this, hence deeming miR-30c-5p “detectable” after intestinal digestion. On the other hand, the ICts of miR-92a-3p in the digests were lower than the 8.92 in the blanks, rendering it “undetectable”.

The obtained results indicated differing stability of the miRs under gastrointestinal conditions. Even though the expression levels of the miRs significantly decreased following gastrointestinal digestion, the current study demonstrates that miR-30c-5p is relatively stable compared to miR-92a-3p. This different stability of miRs has been reported in some other foods [63]. Cavallini et al. [34] detected only 4 out of the starting 22 miRs after intestinal digestion. MiRs do not exhibit this characteristic under gastrointestinal conditions alone. Also, Smyczynska et al. [64] reported an uneven loss of miR-29 and miR-30d-5p families in human breastmilk after thermal treatment, with the latter being the most stable. Hence, this study further demonstrates the stability of another member of the miR-30 family.

Furthermore, the stability of miRs, even plant derivatives [18,19,20,21], strongly supports the hypothesis that honey miRs, after their assumption, are also absorbed into the blood and distributed in body tissues to perform their biological function, namely the regulation of human target mRNA translational processes. This supposition would explain some of the medicinal properties of this natural matrix [18,19,20,21] and suggest new putative biological applications for this food in the immuno-nutrition field.

## 5. Conclusions

The results of our study provide insights into the existence and varying digestive stability of miRs given the same food matrix. We identified in honey the presence of two broadly conserved miRs, miR-30c-5p and miR-92a-3p. Despite honey exhibiting high digestibility, miR-30c-5p demonstrated considerable resistance under gastrointestinal conditions while miR-92a-3p was less resilient. Although the heterogeneity of the nanoparticle profile of honey was influenced by pasteurization treatment, the expression and stability of the miRs remained relatively unchanged. Although there are still many challenges to be solved, such as the absorption, stability, availability, and epigenetic roles of these dietary miRs, the above-discussed results may represent a starting point for the possibility of utilizing dietary miRs from honey for preventive strategies and more “natural” treatments. The field of dietary miRs is still evolving, and further studies and efforts are needed to fully understand their significance and their potential impact on human health.

## Figures and Tables

**Table 1:** Percentage soluble fraction of polyfloral honey, regarding the digestion phase. SSF = simulated salivary fluid.

Digestion Phase	Pasteurized	% Soluble Fraction	
		With Human Saliva	With SSF
**Oral**	Yes	96.32	92.05
	No	96.57	94.29
	Blank	99.22	96.78
**Gastric**	Yes	97.23	94.77
	No	97.41	96.70
	Blank	97.91	97.21
**Intestinal**	Yes	98.95	95.92
	No	98.27	96.84
	Blank	97.41	96.94

**Table 2:** Total RNA Extracted from Polyfloral Honey.

Pasteurization ^1^	µg/g Sample	A260/A280	A260/A230
**Manual Extraction**			
Yes	0.64	1.55	0.7
No	0.51	1.60	0.5
**Semi-Automatic** **Extraction ^2^**			
Yes	9.78	1.79	1.93
No	11.59	1.81	2.01

^1^ No significant difference between pasteurized and unpasteurized samples (independent *t*-test, *p* < 0.05) ^2^ Average of 3 independent extractions.

**Table 3:** Total RNA concentration after digesting intestinal phase using semi-automated extraction method.

State	Pasteurized	Phase	µg/g	A260/A280	A260/A230
**Digested**	Yes	Intestinal	903	1.85	2.10
	No	Intestinal	922	1.86	2.17

**Table 4:** Comparison of miR-30c-5p and miR-92a-3p expression levels. Means ± SD of 2 independent extractions. *p* < 0.05.

miRNA	Pasteurized ^1^	Manual ^2^ (n = 4)	Semi-Automated ^3^ (n = 4)	*p*-Value
**miR-30c-5p**	Yes	32.96 ± 0.71	30.74 ± 1.20	0.154
	No	32.11 ± 0.13	30.81 ± 0.57	0.067
**miR-92a-3p**	Yes	33.60 ± 0.03	30.76 ± 0.06	<0.001
	No	31.94 ± 1.35	29.78 ± 0.39	0.162

^1^ No significant difference according to the pasteurization state; ^2^ in-column extraction kit containing phenol; ^3^ phenol-free extraction using binding beads technology.

## Data Availability

The data that support the findings of this study are available from the corresponding author upon reasonable request.

## Supplementary Materials

The following supporting information can be downloaded at https://www.mdpi.com/article/10.3390/cimb46070443/s1: Figure S1. Location of the two zones from which the honey samples were obtained.
