# Supplementary material for: Differential Digestive Stability of Food-Derived microRNAs: The Case of miR-30c-5p and miR-92a-3p in Polyfloral Honey
Source: Curr Issues Mol Biol. 2024 Jul 15;46(7):7473–85. doi: 10.3390/cimb46070443 (PMC11276035; doi:10.3390/cimb46070443)
Supplement: Supplementary file 1 [file cimb-46-00443-s001.zip › cimb-3109646-supplementary.pdf]

Differential digestive stability of food-derivate microRNAs: the case of miR-30c-5p and miR-92a-3p in polyfloral honey

Diana Marisol Abrego-Guandique [1,†], Olubukunmi Amos Ilori [2,†], Maria Cristina Caroleo [1,3], Roberto Cannataro [3,4], Erika Cione [2,3,*] and Paola Tucci [2]

1 Department of Health Sciences, University of Magna Graecia Catanzaro, 88100 Catanzaro, Italy; dianamarisol.abregoguandique@unicz.it (D.M.A.-G.); mariacristina.caroleo@unicz.it (M.C.C.)
2 Department of Pharmacy, Health and Nutritional Sciences, University of Calabria, 87036 Rende, Italy; lrilkn95a09z335g@studenti.unical.it (O.A.I.); paola.tucci@unical.it (P.T.)
3 Galascreen Laboratories, University of Calabria, 87036 Rende, Italy; rcannataro@nutrics.it
4 Research Division, Dynamical Business & Science Society, DBSS International SAS, Bogota 110861, Colombia
* Correspondence: erika.cione@unical.it; Tel.: +39-0984493147
† These authors contributed equally to this work.

Supplementary Materials

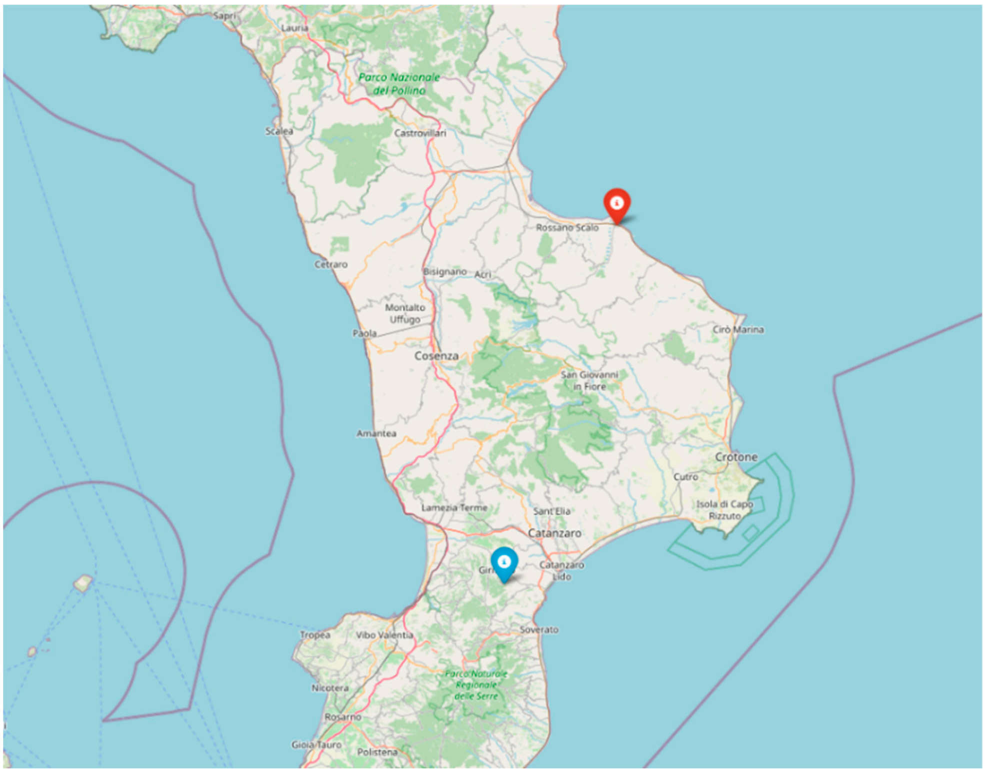

Figure S1. Location of the two zones from which the honey samples were obtained. Folium (v. 0.15.1) on Python software (v 3.9.12).
